# Arsenite-Mediated Transcriptional Regulation of Glutathione Synthesis in Mammalian Primary Cortical Astrocytes

**DOI:** 10.3390/ijms26115375

**Published:** 2025-06-04

**Authors:** Jacob P. Leisawitz, Jiali He, Caroline Baggeroer, Sandra J. Hewett

**Affiliations:** Interdisciplinary Neuroscience Program, Department of Biology, Syracuse University, Syracuse, NY 13210, USA; jacob.leisawitz@bcm.edu (J.P.L.); jiali_he@urmc.rochester.edu (J.H.); caroline.e.baggeroer@vanderbilt.edu (C.B.)

**Keywords:** astrocytes, arsenicals, glutathione, oxidative stress, amino acid transport systems, glutathione-cysteine ligase, glutathione synthetase, transcription, genetic, blood–brain barrier, RNA, messenger

## Abstract

Arsenic, a potent metalloid contaminant of drinking water, is known for its ability to act as an initiator and modulator of disease in a variety of human tissues. Upon ingestion, arsenic is bio-transformed in the liver into a variety of metabolites, including arsenite. Arsenite permeates the blood–brain barrier (BBB), inducing oxidative stress that can be detrimental to brain neurons. As the primary glial cell at the BBB interface, astrocytes play a pivotal role in detoxifying xenobiotics such as arsenite via the production of the tripeptide antioxidant γ-glutamylcysteine, or glutathione (GSH). In this study, we assessed the mRNA levels of key components of the GSH synthetic pathway in astrocytes exposed to arsenite compared to vehicle controls. These components included xCT [substrate-specific light chain of the substrate importing transporter, system x_c_^−^ (Sx_c_^−^)], glutamate-cysteine ligase [both catalytic (GCL_C_) and modifying (GCL_M_) subunits], and glutathione synthetase (GS). Additionally, we analyzed protein levels of some components by Western blotting and evaluated functional activity of Sx_c_^−^ using a fluorescence-based cystine uptake assay. Finally, we utilized a luminescence-based glutathione assay to determine the intracellular and extracellular GSH content in arsenite-treated cells. Arsenite significantly increased xCT, GCL_C_, GCL_M_, and GS mRNA levels, an effect blocked by the transcriptional inhibitor actinomycin D (ActD). A corresponding increase in Sx_c_^−^ activity was also observed in the arsenite treatment groups, along with significant increases in GCL_C_ and GCL_M_ protein expression. However, no increase in GS protein expression was detected. Finally, arsenite treatment significantly increased extracellular GSH levels, an effect which was also prevented by the inclusion of ActD. Overall, our study provides evidence that arsenite transcriptionally regulates several cellular processes necessary for GSH synthesis in primary cortical astrocyte cultures, thereby contributing to a better understanding of how this environmental toxicant influences antioxidant defenses in the brain. However, these results should be interpreted with caution regarding their applicability to vivo systems.

## 1. Introduction

Arsenic is a toxic metalloid found in abundance in the earth’s crust that is released into the biosphere by both natural and anthropogenic processes [1]. Arsenic contamination of ground water poses a serious public health risk to humans around the world. In some regions, groundwater arsenic concentrations can exceed 50–100 µg/L—far above the World Health Organization’s recommended limit of 10 µg/L (10 ppb) [2,3]. While high levels of arsenic in drinking water are most prominent in countries such as Bangladesh and Taiwan, concentrations of over 3000 µg/L have been discovered in wells in the United States [4]. Exposure to this metalloid has been implicated in the pathogenesis of various maladies such as cardiovascular disease, renal disease, and several neurological impairments [5,6]. When arsenic enters the body, it undergoes biotransformation into a wide range of organic and inorganic metabolites, including arsenite [7]. Arsenite is particularly dangerous to the central nervous system (CNS) because it can cross the blood–brain barrier (BBB) [8], which normally impedes harmful substances from entering the brain [9]. The BBB is composed of various elements of the neurovascular unit (NVU), highlighting the close interplay between the basal lamina, endothelial cells of the cerebrovasculature, pericytes, and glial cells, which collectively regulate the flow of substances between the bloodstream and the brain parenchyma [10].

Among glial cells, astrocytes are the predominate type in the CNS [11] and are an integral component of the BBB [12]. Astrocyte dysfunction has been implicated in multiple CNS disorders, including neurodegeneration, stroke, and neuroinflammation, emphasizing their central role in CNS homeostasis (for a review, see [13,14]). Indeed, astrocytes perform a variety of functions, including but not limited to the facilitation of neuronal migration, regulation of the extracellular environment, modulation of neurotransmitter levels in the synaptic cleft, formation and maintenance of the BBB, and detoxification of xenobiotics [15,16]. Given their role in maintaining the BBB, astrocytes are the first line of defense against toxicants that enter the brain, such as arsenite [17]. One of their more important protective functions is defending against oxidative stress, primarily through the production of glutathione (GSH; for a review, see [18]). Exposure of cultured astrocytes to millimolar levels of arsenite for just a few hours results in a loss of cellular GSH content [17,19]. In contrast, when exposed to low micromolar levels of arsenite, astrocytes exhibit an elevation in GSH levels [17].

GSH is one of the most important antioxidants in the body, owing its ability to maintain cellular redox homeostasis due to its free sulfhydryl group [20]. Astrocytes have been shown to produce high concentrations of GSH [21], exporting it at a rate of 10% per hour [22]. This export provides neurons with the necessary substrates for their own GSH synthesis [21,23,24]. The GSH synthesis process involves two reactions catalyzed by two unique ATP-consuming enzymes: glutamate-cysteine ligase (GCL) and glutathione synthetase (GS), requiring three substrates: cysteine, glutamate, and glycine [25]. Cysteine is the rate-limiting substrate for the production of GSH [26]. In astrocytes, it is supplied by the import of cystine via a heterodimeric amino acid antiporter, system x_c_^−^ (Sx_c_^−^; for a review, see [27]). Upon import, cystine is reduced to cysteine. GCL, composed of a catalytic (GCL_C_) and modifier (GCL_M_) subunit, then carries out the synthesis of the dipeptide γ-glutamylcysteine from glutamate and cysteine. Finally, GS adds the amino acid glycine to generate the final tripeptide product (for a review, see [25]). Once synthesized, GSH can be utilized intracellularly and/or be released from astrocytes into the extracellular space through various mechanisms [19,23,24,28,29,30,31,32,33].

This study seeks to determine how arsenite exposure impacts GSH synthesis in astrocytes. Specifically, we explore whether exposure of primary cortical astrocytes to low, non-toxic concentrations of arsenite regulates the expression of key enzymes and transporters responsible for GSH biosynthesis.

## 2. Results

### 2.1. Effect of Arsenite on Astrocyte GSH Levels

**Figure 1:**
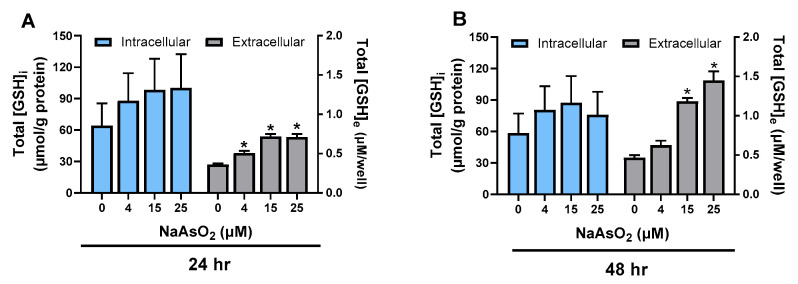
***Effect of arsenite on astrocyte GSH levels.*** Primary cortical astrocytes (*n* = 6–9 from 2–3 separate dissections) were treated with increasing concentrations of NaAsO_2_ for 6 h. The NaAsO_2_ was removed by washing, and the cultures were incubated for either 24 h (**A**) or 48 h (**B**). Data are expressed as mean µmol/g protein + SEM normalized to protein content in each individual well (intracellular) or mean µM/well + SEM (extracellular). Intracellular GSH levels were analyzed by the Kruskal–Wallis test followed by Dunn’s test for multiple comparisons. Extracellular GSH concentrations were analyzed by ordinary one-way ANOVA followed by Šídák’s test for multiple comparisons. No significant changes in intracellular levels were observed between any groups at either time point. An asterisk (*) denotes extracellular concentration values significantly different from untreated cells (0µM) only [(**A**) 0 vs. 4 µM, *p* = 0.0049; 0 vs. 15 µM, *p* < 0.0001; 0 vs. 25 µM *p* < 0.0001; (**B**) 0 vs. 15, *p* < 0.0001; 0 vs. 25 *p* < 0.0001].

Exposure of primary cortical astrocytes to non-cytotoxic concentrations of arsenite (Supplemental Figure S1) did not result in significant changes in intracellular ([GSH]_i_) levels. However, increases in the accumulation of GSH in the extracellular medium ([GSH]_e_) occurred in a concentration-dependent manner, measured 24 h and 48 h post-exposure (Figure 1A,B). Our data indicate that this effect reached a plateau at around 15 µM, with no significant differences observed between 15 µM and 25 µM at 24 (*p* > 0.9999) and 48 h (*p* = 0.0595), respectively. Based on these findings, we selected 15 µM as the optimal concentration for all subsequent experiments.

### 2.2. Effect of Arsenite on Astrocyte xCT mRNA Expression and Transporter Activity

**Figure 2:**
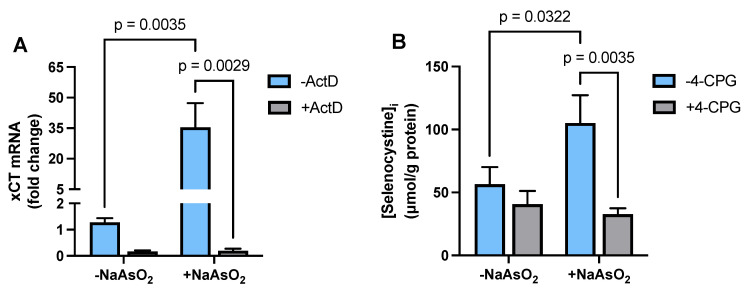
***Effect of arsenite on astrocyte xCT mRNA expression and transporter activity.*** (**A**) Primary cortical astrocytes (*n* = 3 from 3 separate dissections) were treated with 10 µg/mL ActD (+ActD; gray bars) or its vehicle (−ActD; blue bars). Thirty minutes later, NaAsO_2_ was spiked into experimental wells to a final concentration of 15µM. After 4 h, RNA was isolated, and xCT mRNA expression was determined by RT-qPCR. Data are expressed as mean + SEM fold change in xCT mRNA over untreated cells (−NaAsO_2_, −ActD; =1). (**B**) Primary cortical astrocytes (*n* = 4 from separate dissections) were treated with NaAsO_2_ (15µM) for 24 h. Selenocystine (25 µM) was added to the cultures in the absence (−4-CPG, blue bars) or presence (+4-CPG, gray bars) of 4-CPG (500 µM) for 30 min. Cells were washed in methanol to permeabilize their plasma membranes, after which cellular extracts were collected and selenocystine concentration quantified, as described in the Section 4. Exact *p*-values are shown for each pairwise comparison that was determined to be significant by two-way ANOVA followed by Fisher’s uncorrected LSD test for multiple comparisons.

To determine whether arsenite regulated cysteine availability, we measured mRNA expression levels of xCT, the substrate-specific light chain of Sx_c_^−^, and Sx_c_^−^ functional activity. Exposure of primary cortical astrocytes to 15 µM arsenite resulted in a significant increase in steady-state xCT mRNA expression (Figure 2A). Treatment with the transcriptional inhibitor actinomycin D (ActD, 10 µg/mL) prevented this arsenite-induced increase in xCT mRNA (Figure 2A). Consistent with the qPCR data, we found that selenocystine uptake into astrocytes was significantly increased following arsenite exposure. The inclusion of the Sx_c_^−^ competitive inhibitor, 4-carboxyphenylglycine (4-CPG) [34], confirmed that the observed increase in selenocystine uptake was mediated by Sx_c_^−^ (Figure 2B).

### 2.3. Effect of Arsenite on Astrocyte GCL mRNA and Protein Expression

**Figure 3:**
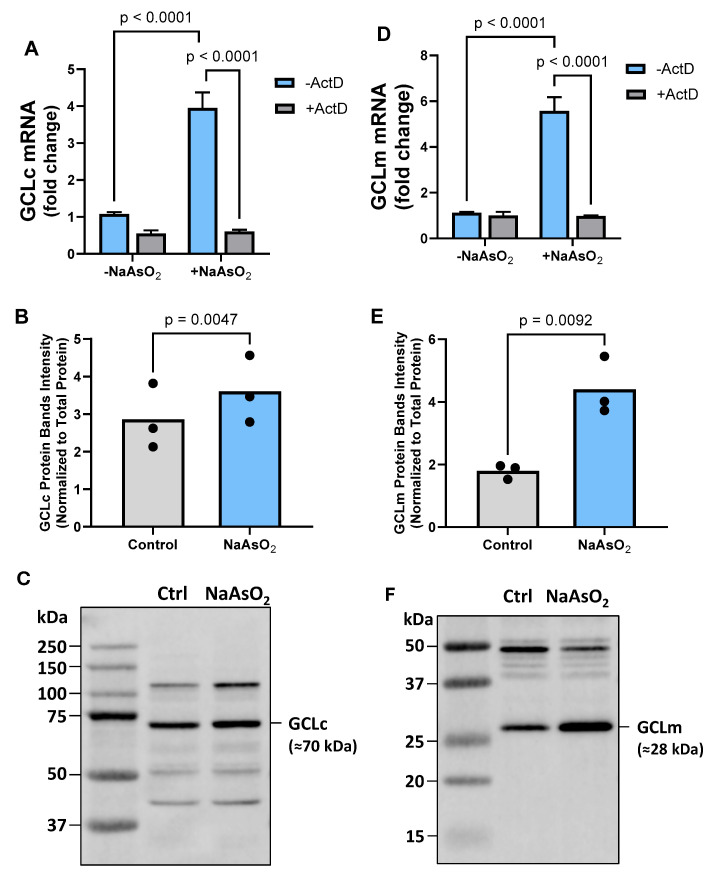
***Effect of arsenite on astrocyte GCL mRNA and protein expression.*** Primary cortical astrocytes (*n* = 3 from 3 separate dissections) were treated with 10 µg/mL ActD (+ActD; gray bars) or its vehicle (−ActD; blue bars) for 30 min, after which NaAsO_2_ was spiked into experimental wells to a final concentration of 15µM. Four hours later, RNA was isolated, and (**A**) GCL_C_ and (**D**) GCL_M_ mRNA expression was determined by RT-qPCR. Data are expressed as mean + SEM fold change over untreated cells (−NaAsO_2_, −ActD; =1). Exact *p*-values are shown for each pairwise comparison determined by two-way ANOVA followed by Fisher’s uncorrected LSD test for multiple comparisons. Protein expression of (**B**) GCL_C_ and (**E**) GCL_M_ was assessed by Western blotting, as described in the Section 4. Exact *p*-values are shown for each pairwise comparison determined to be significant by a paired *t*-test. Representative blots for (**C**) GCL_C_ and (**F**) GCL_M_ are shown.

While increased substrate availability alone could potentially explain the enhanced GSH accumulation observed in astrocyte medium upon arsenite exposure [35], we next sought to explore the role of other key components of the GSH synthetic pathway. First, we assessed mRNA expression for glutamate-cysteine ligase (GCL), the enzyme that catalyzes the first committed step of GSH synthesis [20,25,36]. Astrocytes treated with 15µM arsenite experienced an increased steady-state mRNA level of both the catalytic (GCL_C_) and modifying (GCL_M_) subunit of GCL. Importantly, this upregulation was found to be transcriptionally mediated (Figure 3A,D). To determine whether the increased mRNA levels led to corresponding increases in protein expression, we performed a Western blot analysis. As shown in Figure 3B,E, we observed a significant increase in the protein levels of both GCL subunits, confirming that the elevated mRNA was indeed translated into higher protein expression.

### 2.4. Effect of Arsenite on Astrocyte GS mRNA and Protein Expression

**Figure 4:**
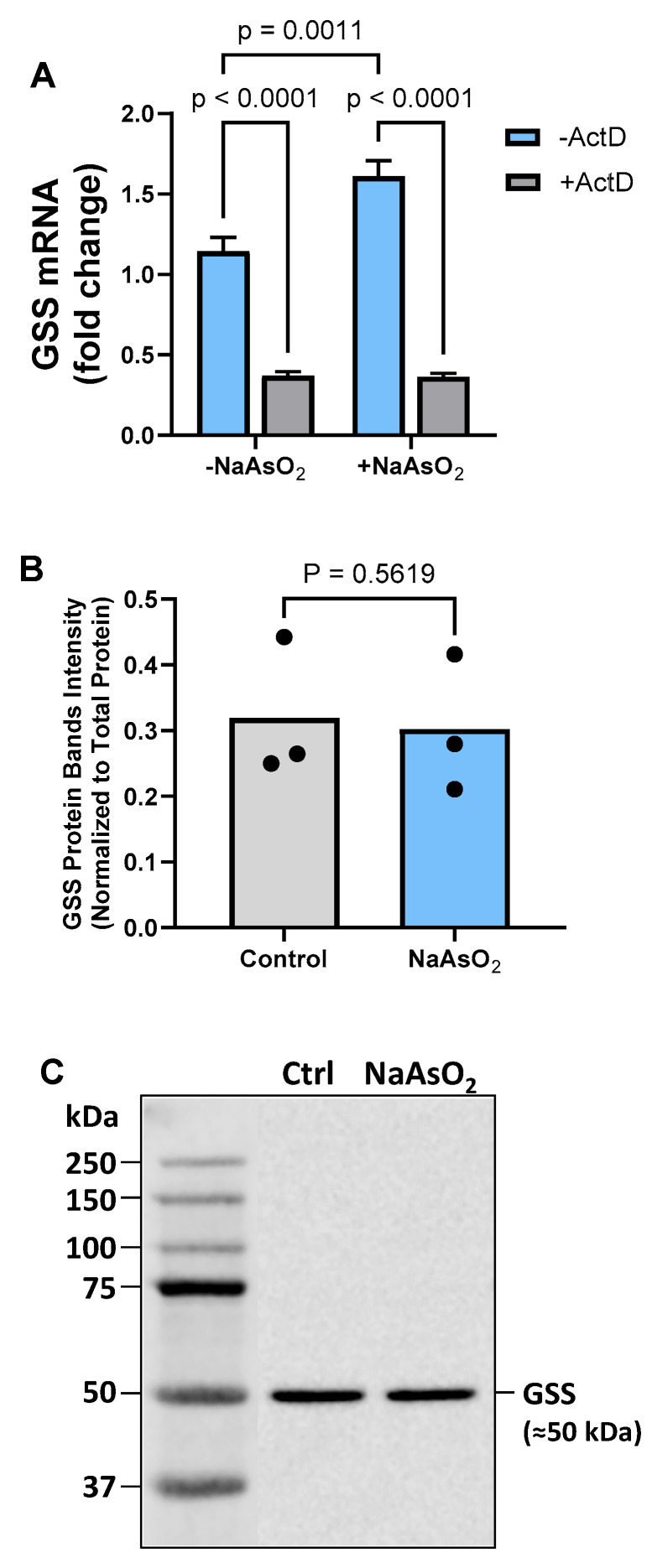
***Effect of arsenite on astrocyte GS mRNA and protein expression.*** (**A**) Primary cortical astrocytes (*n* = 3 from 3 separate dissections) were treated with 10 µg/mL ActD (+ActD; gray bars) or its vehicle (−ActD; blue bars) for 30 min, after which NaAsO_2_ was spiked into experimental wells to a final concentration of 15µM. Four hours later, RNA was isolated, and GS mRNA expression was determined by RT-qPCR. Data are expressed as mean + SEM fold change in GS mRNA over untreated cells (−NaAsO_2_, −ActD). Exact *p*-values are shown for each pairwise comparison that was determined to be significant, as assessed by two-way ANOVA followed by Fisher’s uncorrected LSD test for multiple comparisons. (**B**) Protein expression of GS was assessed by Western blotting, as described in the Section 4. No significant changes in GS protein were determined by a paired *t*-test. (**C**) Representative blot for GSS.

Next, we explored the effect of arsenite exposure on mRNA levels of GS, the enzyme that catalyzes the second and final committed step of the GSH synthetic pathway. Astrocytes exposed to 15 μM arsenite showed a slight but significant transcriptionally dependent increase in GS mRNA expression (Figure 4A). However, no increase in protein expression was found (Figure 4B).

### 2.5. Transcriptional Regulation of Astrocytic GSH Levels by Arsenite

**Figure 5:**
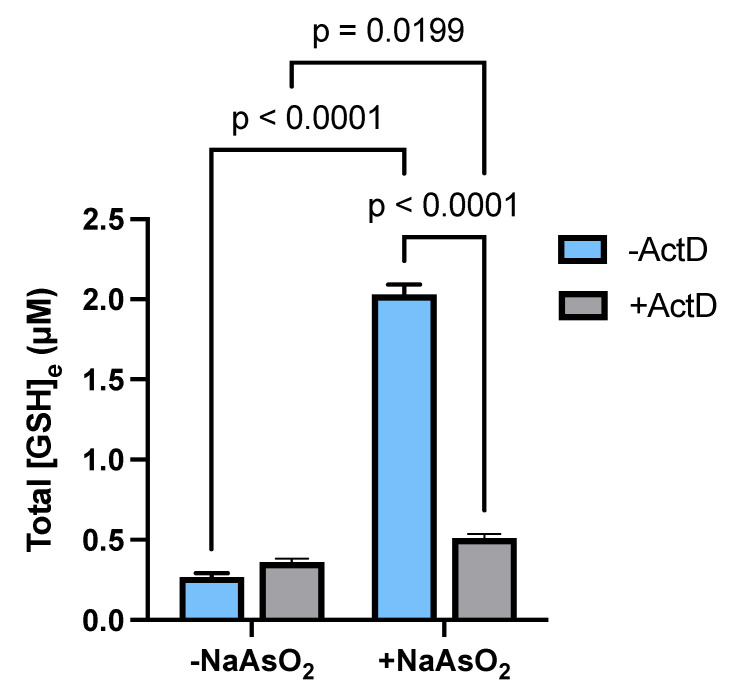
***Transcriptional regulation of astrocytic GSH levels by arsenite*.** Primary cortical astrocytes (*n* = 5–6 from one dissection) were treated with ActD (10 µg/mL) or its vehicle for 30 min. NaAsO_2_ was then spiked into experimental wells (final concentration = 15 µM). A total of 6 h later, arsenite and/or ActD were removed by thorough washing, and cells were incubated for 48 h in phenol-red-free MS supplemented with L-glutamine. Aliquots of experimental medium were pulled for measurement of extracellular levels of GSH. Data are expressed as mean µM total GSH + SEM. Exact *p*-values are shown for each pairwise comparison that was determined to be significant, as assessed by two-way ANOVA followed by Fisher’s uncorrected LSD test for multiple comparisons.

Taken together, our data indicates that arsenite upregulates key components of the GSH synthetic pathway primarily through transcriptionally dependent mechanisms. Therefore, we hypothesized that inhibiting transcription would prevent the observed arsenite-induced increase in extracellular GSH accumulation. Indeed, treatment of the astrocyte cultures with ActD (10 µg/mL) blocked [GSH]_e_ accumulation in media from astrocytes exposed to 15 µM arsenite (Figure 5).

## 3. Discussion

Upon ingestion, inorganic arsenicals, such as arsenite, can cause neurological dysfunction, as they readily penetrate the BBB [8]. Due to their strategic positioning along the cerebrovasculature, arsenite initially interacts with astrocytes. Our investigation demonstrates that exposing primary astrocytes to arsenite leads to increased GSH accumulation in the extracellular medium, without any significant alterations in intracellular GSH levels. These results are consistent with previous findings [17], which show that low micromolar levels of arsenite increase extracellular GSH levels without depleting intracellular stores, as is observed when arsenite is given in higher (millimolar) concentrations [19,37]. The ability of the astrocytes to maintain intracellular levels despite significant release into the extracellular space suggests a finely tuned regulatory mechanism governing GSH synthesis and release. One possible explanation is that astrocytes possess robust mechanisms for replenishing intracellular GSH levels promptly after release. In support of this idea, our data demonstrate that exposure of astrocytes to arsenite upregulates mRNA production of the substrate importing transporter, Sx_c_^−^, and the GSH synthetic enzymes GCL and GS. Further, our study provides evidence for the arsenite-induced increase in activity of Sx_c_^−^, as well as an increase in protein expression of GCL_C_ and GCL_M_ but not GS.

With respect to substrate import, previous studies in our laboratory found that the half-life of xCT mRNA in arsenite exposed astrocytes was not significantly different from the control, indicating that post-transcriptional mechanisms were not the primary mode of regulation (Supplemental Figure S2). Instead, our current study reveals that arsenite induces an increase in steady-state xCT mRNA expression levels via a process requiring transcription. This increase in xCT mRNA expression appears to be functionally important, as it is accompanied by a significant increase in selenocystine uptake compared to vehicle-treated controls. This increased uptake is abolished by 4-CPG, an inhibitor of Sx_c_^−^ [34,38,39,40]. While 4-CPG does not fully eliminate basal uptake—likely due to the presence of additional cystine and cysteine transport systems in astrocytes [41,42]—its ability to block the arsenite-mediated increase provides strong evidence that the elevated uptake is primarily driven by increased Sx_c_^−^ activity.

Transcription, a process that copies DNA to RNA, relies on the recruitment of transcription factors that bind to specific DNA sequences, thereby initiating and regulating gene expression. Although we did not elucidate the exact cis and trans factors that mediate transcription of xCT in our system, insights from the literature offer potential avenues for further exploration. Arsenite has been shown to effectively permeate the BBB and induce oxidative stress via the formation of free radicals and reactive oxygen species (ROS), including hydrogen peroxide, hydroxyl radicals, and superoxide anions [43]. Among the mechanisms implicated in the cellular response to oxidative stress are electrophilic response elements (EpREs), also known as antioxidant response elements (AREs). Notably, four EpRE-like sequences [EpREs (1-4)] have been identified in the 5′ flanking region of the xCT gene [44]. Mutational analysis of EpRE-1 within the xCT gene has demonstrated its significant involvement in the transcriptional regulation of xCT in BHK21 fibroblasts when cells were exposed to diethyl maleate (DEM) and, importantly, to arsenite [44].

Binding to these ARE/EpRE sites is the transcription factor nuclear factor erythroid 2-related factor 2 (Nrf2). Nrf2 becomes activated under oxidative stress conditions, such as is known to occur in the presence of arsenite [45]. Nrf2 is known to regulate GSH metabolism [46,47], and there is ample evidence for the involvement of this transcription factor in the regulation of xCT expression in a variety of different tissues in both positive and negative directions [27,48,49,50,51,52]. Interestingly, meningeal tissue derived from Nrf2-knockout mice showed a decreased expression of xCT compared to wild-type littermates, as assessed by Western blotting [52]. Additionally, increased xCT expression has been shown to be dependent on Nrf2 in astrocytes treated with amyloid-beta fragments [53]. Finally, pyridoxine-induced activation of Nrf2 has been shown to increase GSH production in astrocytes [54] as well as in astrocytoma U373 cells [55].

Despite robust evidence for the role of Nrf2 in regulating xCT, previous studies in our lab have shown that Nrf2 is not required for arsenite-induced upregulation of xCT mRNA in astrocytes (Supplemental Figure S3). This raises questions about the ways that arsenite induces cellular stress, which signaling pathways and transcription factors those stressors activate, and the downstream genes whose expression patterns are altered as a result. Although oxidative stress is one of the primary mechanisms by which arsenite induces cellular stress [17,43], it is conceivable that arsenite might trigger xCT expression through alternate pathways. These could include genotoxicity [56,57] and/or protein misfolding/aggregation, which would engage distinct cell signaling pathways that regulate xCT expression via transcription factors other than Nrf2. For instance, genotoxic stress is known to activate activator protein-1 [AP-1 (c-Fos:c-Jun)] [58,59] and nuclear factor kappa-B [NF-kB, (p50:p65)] [60,61]. Pertinently, both sites are present on the xCT gene [62]. Additionally, knockout of activating transcription factor 4 (ATF4) [63] reduced, but did not eliminate, the expression of xCT mRNA in murine hepatocytes transformed to hepatocellular carcinoma (HCC) cells [50], implicating the involvement of the amino acid response elements (AAREs) in mediating xCT expression.

The present study also provides evidence for transcriptional regulation of both subunits of GCL. GCL_C_ is the catalytic subunit of the enzyme, whereas GCL_M_ modulates GCL_C_ activity by increasing its V_max_ and k_cat_ [64], thereby allowing for optimal activity of the holoenzyme. Similar to our findings, arsenite exposure of murine hepatocytes transcriptionally regulated both GCL_C_ and GCL_M_ expression [65]. GCL_M_ was also regulated via post-transcriptional stabilization [65], something not explored herein. Interestingly, in these hepatocytes, the arsenite-mediated increase in both subunits of GCL occurred via Nrf2-independent mechanisms [65]. Likewise, research in mouse embryonic fibroblasts, comparing cells derived from Nrf2-null mice to wild-type mice, found no significant difference in arsenite-induced GCL_C_ and GCL_M_ mRNA expression [65]. ATF4 might also be ruled out, as knockout had no significant effect on expression levels of GCL_C_ and GCL_M_ in hepatocellular carcinoma cells [50]. Interestingly, p38 mitogen-activated protein kinase (MAPK) and extracellular signal-related kinase (ERK) signaling pathways regulate arsenite-induced expression of GCL subunits in TGF-α-overexpressing mouse hepatocytes (TAMH) [66]. The activation of these signaling pathways is regulated by various transcription factors, including activating transcription factor-2 (ATF-2), AP-1, and NF-kB [67,68,69]. Whether these signaling pathways are involved in the arsenite-mediated increase in GCL in astrocytes remains to be determined.

Regardless of the mechanism by which this occurs, our data shows a 4–6-fold increase in GCL_C_ and GCL_M_ mRNA in cells treated with 15µM arsenite. Given that GCL is the rate-limiting enzyme for the GSH synthetic pathway [25], one might expect a significant increase in intracellular GSH concentrations following arsenite exposure given the significant upregulation of its subunits at both the mRNA and protein levels. Despite this, our data shows no significant changes in intracellular GSH levels induced by arsenite exposure. This may be due to the operation of a negative feedback loop, whereby GSH inhibits its own synthesis by slowing the function of GCL [70,71]. Thus, once sufficient levels of GSH are restored following arsenite-mediated export, GSH inhibits GCL function to prevent excessive synthesis. Since GCL requires ATP to synthesize the dipeptide γ-GluCys [25], it makes sense that the cell would tightly regulate the function of this enzyme once sufficient intracellular stores are replenished so as to not deplete ATP stores unnecessarily. It may also simply be that synthesis matches release.

Despite the robust arsenite-induced upregulation of GCL subunits, our data shows that GS mRNA is only slightly, though significantly, upregulated via transcriptional mechanisms. However, this does not result in an increase in GS protein expression, as determined by immunoblotting. One possible explanation is that GS mRNA is transcribed at a rate comparable to GCL but undergoes more rapid cytosolic degradation, limiting its accumulation and subsequent translation. Alternatively, the observed transcriptional upregulation may be insufficient to drive a corresponding increase in protein levels due to translational or post-translational regulatory controls. Future studies will be necessary to investigate whether GS protein expression is constrained by mRNA stability, translational efficiency, or protein turnover in this context. Finally, it should be noted that GS has a higher substrate affinity and is not as tightly regulated as GCL [25,72]. Thus, astrocytes may not need elevated levels of GS to maintain GSH levels, as the availability of γ-glutamylcysteine produced by increased GCL activity could potentially satisfy the demand for GSH synthesis. However, coordinated expression of GCL and GS is known to occur in other cell types [73,74].

In conclusion, our study provides compelling evidence that arsenite upregulates key processes essential for GSH synthesis in primary cortical astrocyte cultures in a transcription-dependent manner, including Sx_c_^−^, GCL_C_, and GCL_M_. Taken together, our findings suggest that these components contribute, either individually or via coordinated mechanisms, to the observed increase in GSH accumulation in the media of astrocyte cultures exposed to arsenite. These results contribute to a better understanding of how the environmental toxicant arsenite influences antioxidant defenses in the brain. While this study is limited to in vitro experiments in isolated astrocytes, these controlled conditions enabled us to uncover specific mechanistic insights into arsenite-mediated gene regulation. Importantly, these findings establish a foundation for future in vivo studies aimed at understanding the cellular and molecular basis of arsenic neurotoxicity in the context of the intact neurovascular unit and the complex CNS microenvironment.

## 4. Materials and Methods

### 4.1. Experimental Media and Buffers

Cell culture media and experimental buffer compositions were as follows:

- *Media Stock (MS)*: Minimum Essential Media with Earle’s salts purchased without L-glutamine (Corning, Corning, NY, USA) supplemented with L-glutamine (L-gln; Gibco, Burlington, ON, Canada), glucose, and sodium bicarbonate to a final concentration of 2.0, 25.7, and 28.2 mM, respectively, and stored at 4 °C. This MS, which contained 100 µM cystine, as provided in the base MEM formulation, served as the foundation for all other cell culture media used in this study.
- *Astrocyte Plating Media (APM)*: MS was supplemented with 10% Calf Serum (CS; Hyclone Laboratories, Marlboro, MA, USA), 10% Fetal Bovine Serum (FBS; Hyclone), 50 IU penicillin, and 50 µg/mL streptomycin (Gibco) and stored at 4 °C. L-gln and epidermal growth factor (EGF; Thermo Fischer Scientific, Waltham, MA, USA) were added to a final concentration of 2 mM and 10 ng/mL, respectively. Medium was warmed to 37 °C immediately prior to use.
- *Maintenance Media-1 (MM-1)*: MS was supplemented with 10% CS, 50 IU penicillin, and 50 µg/mL streptomycin and stored at 4 °C. L-gln was supplemented to a final concentration 2 mM. Medium was warmed to 37 °C just prior to use.
- *Maintenance Media*-*2 (MM-2)*: MS was supplemented with 3% CS, 50 IU penicillin, and 50 µg/mL streptomycin and stored at 4 °C. L-gln was supplemented to a final concentration 2 mM. Medium was warmed to 37 °C just prior to use.
- *1*× *BSS_10_*: Cell-culture-grade water (Corning) containing 116.4 mM sodium chloride (NaCl; Thermo Fischer Scientific), 5.4 mM potassium chloride (KCl; Thermo Fischer Scientific), 0.8 mM magnesium sulfate heptahydrate (MgSO_4_·7H_2_O; Sigma-Aldrich, St. Louis, MO, USA), 1 mM sodium phosphate monobasic monohydrate (NaH_2_PO_4_·H_2_O; Thermo Fischer Scientific), 10 mM glucose, and 26 mM sodium bicarbonate was used. A 10× stock was generated and filter-sterilized before being stored at room temperature. A 1× BSS_10_ solution was then generated from the stock solution with sterile cell-culture-grade water in a sterile environment. Just prior to use, 1.8 mM CaCl_2_ was slowly added for a duration of five minutes while CO_2_ was bubbled into the solution. The 1× BSS_10_ was also stored at room temperature.

### 4.2. Astrocyte Cultures

Primary cortical astrocytes were cultured from cortices of 1–3-day postnatal CD1 mice (Charles River Laboratories, Wilmington, MA, USA). Cultures were established by seeding dissociated cells on 6- or 24-well tissue culture plates (Corning, Primaria) at a density of 3–4 hemispheres/10 mL APM/plate. Subsequently, the astrocytes were allowed to reach confluence, following which they were treated once with 8µM cytosine β-D-arabinofuranoside (Ara-C). Under these conditions, AraC selectively inhibited the proliferation of rapidly dividing contaminating cells, such as microglia, while having minimal impact on the now-quiescent astrocyte population. After 6 to 7 days of exposure to Ara-C, the astrocyte culture medium was fully replaced with MM, which was then refreshed weekly. This plating method consistently yielded confluent monolayers of cells with a protoplasmic morphology, which were previously confirmed to be astrocytes based on their immunoreactivity to an antibody against glial fibrillary acidic protein (GFAP) [75]. One day prior to experimentation, the astrocyte cultures were washed in MM containing 3% CS. This step served as a transition to the fully serum-free media used for all subsequent experimental treatments. All cultures were maintained in an incubator at 37 °C, 5.5% CO_2_, and 21% O_2_. Cultures that exceeded 35 days in vitro (DIV) were excluded from experimentation.

### 4.3. Drug Stocks and Exposures

- *Arsenite (Na_2_AsO_2_)*: Arsenite stock (25 mM) was prepared by dissolving sodium arsenite (Sigma-Aldrich) in MS and adjusting the pH to 7.0 using 1.65 N HCl. This stock solution was stored at 4 °C. Arsenite sub-stocks were generated by diluting the stock in MS to concentrations of 4, 10, 15, or 25 µM. Micromolar levels of arsenite have been commonly used in vitro to facilitate the identification of potential pathways and targets affected by arsenic, including those involved in the regulation of GSH synthesis [17,37,76]. On the day of experimentation, the astrocyte culture medium was fully aspirated, and cells were washed twice with MS (400 µL or 1.6 mL for 24- or 6-well plates, respectively). Cultures were treated with arsenite by either direct addition of the sub-stocks or by spiking the stock solution into wells to the desired final concentration. The exposure duration lasted 4 to 6 h depending on the specific requirements of the experimental protocol.
- *Actinomycin D (ActD)*: To inhibit transcription, ActD (Cayman, Ann Arbor, MI, USA) was utilized [77]. ActD stock (10 mg/mL) was prepared in DMSO and stored at −20 °C. A sub-stock (10 µg/mL) was made by diluting the stock solution into MS, resulting in a final DMSO concentration of =0.1%. Vehicle controls were prepared by adding DMSO to MS to the same final concentration. Astrocyte cultures were pretreated with either ActD (10 µg/mL) or vehicle (0.1% DMSO) prior to arsenite exposure (15 µM final concentration). The concentration of ActD used in these studies was consistent with prior work by our group and others demonstrating effective transcriptional inhibition while preserving astrocyte viability [78,79,80].
- *4-carboxyphenylglycine (4-CPG)*: 4-CPG (Tocris, Avonmouth, Bristol, UK) was used to competitively inhibit Sx_c_^−^ [34,39,40]. 4-CPG stock (50 mM) was prepared in 1 N NaOH and stored at −20 °C. The stock solution was diluted to a final concentration of 500 µM in 1× BSS_10_ just prior to addition to cultures.
- *Selenocystine (SeCyss)*: SeCyss (Sigma-Aldrich) was utilized as an analog of cysteine to quantitatively measure Sx_c_^−^ activity in astrocytes [81]. SeCyss stock (100 mM) was prepared in 1 N NaOH. The solution was stored at −20 °C. On the day of experimentation, a 25 mM sub-stock was generated in 1N NaOH, which was further diluted in 1× BSS_10_ to a final concentration of 25 µM.

### 4.4. Glutathione Measurement

Cells were washed twice in MS (400 µL or 1.6 mL for 24- or 6-well plates, respectively) before undergoing a 30 min pre-treatment with either ActD or its vehicle, after which arsenite was spiked into appropriate wells to a final concentration of 15µM. After six hours of incubation, the cell culture medium was replaced with phenol-red-free MS supplemented with L-gln (2 mM). This approach ensured that arsenite was removed prior to the assay, eliminating potential interference with the GSH detection assay during the time of measurement. After 24 or 48 h, the medium was collected for analysis of intracellular and extracellular GSH levels. Measurements were carried out using the GSH-Glo glutathione assay (Promega, Madison, WI, USA), as per the manufacturer’s instructions. Luminescence was quantified using a Synergy2 microplate reader (Biotek, Winooski, VT, USA). GSH levels were quantified by measuring the luminescence emitted, calibrated against a standard curve generated from known concentrations of GSH standards prepared in the GSH-Glo reagent. Data are expressed as mean µmol GSH/g protein + SEM (intracellular) or µM GSH + SEM (extracellular).

### 4.5. Reverse Transcription–Quantitative Polymerase Chain Reaction (RT-qPCR)

To capture early transcriptional responses, total RNA was isolated from one well of a six-well plate using 1 mL of TriZol reagent, as per manufacturer’s instructions 4 h after arsenite exposure. First-strand cDNA was synthesized from 0.2–1 µg of RNA using MMLV Reverse Transcriptase (Invitrogen, Waltham, MA, USA) and oligo d(t) primers (Promega) as previously described [82]. The resulting cDNA served as a proxy for mRNA expression in subsequent quantitative polymerase chain reaction (qPCR) analyses. qPCR was performed using TaqMan Universal PCR Master Mix (Fischer, Fautenbach, Germany) and mouse-specific primer pairs (TaqMan Gene Expression Assays, Applied Biosystems, Waltham, MA, USA): xCT (Mm00442530_m1), GAPDH (Mm99999915_m1), GS (Mm00515065_m1), GCL_C_ (Mm00602655_m1), GCL_M_ (Mm00514996_m1), HPRT (Mm01545399_m1), and MRP1 (Mm00456156_m1). The relative quantity of cDNA was calculated via the comparative cycle threshold method (ΔΔC_t_), where cycle threshold values (C_t_) for the gene of interest were normalized to an invariant housekeeping gene, GAPDH, which was confirmed to be stable in the presence of arsenite and/or actinomycin D. C_t_ values from the same samples were then compared with a calibrator sample C_t_ (control group) to determine the relative fold increase in mRNA. Experiments were performed to establish the amplification efficiencies of all primers used. Only primer pairs for the housekeeping gene and gene of interest that exhibited amplification efficiencies within 7% of each other were utilized to ensure accurate quantification.

### 4.6. (Seleno)Cystine Uptake Assay

The uptake of selenocystine into astrocytes was quantified using a fluorescence-based protocol validated to accurately represent transport mediated by Sx_c_^−^ [81]. In short, astrocyte cultures plated in 6-well plates were washed into MS (2×, 1.6 mL), after which cells were incubated with arsenite (15 µM) or its vehicle (each at 1.6 mL). After 24 h of incubation—consistent with the original protocol using diethyl maleate (DEM)—arsenite was removed by thorough washing (3×, 1.6 mL, 1× BSS_10_). A total of 5 min later, 25 µM SeCyss with or without 500 µM 4-CPG (final well volume = 1.6 mL) was washed into the cultures and placed back into the incubator at 37 °C for 30 min. The medium was aspirated, and 2 mL of ice-cold PBS was added directly to each well to arrest cell metabolism. The PBS was then aspirated, and 250 µL of ice-cold 100% methanol was added to permeabilize the cultures, thereby facilitating the release of selenocystine. The plate was promptly covered to prevent methanol evaporation and placed on a rocker for 5 min at 4 °C. Methanol supernatants were diluted 1:1 with H_2_O (Corning) followed by rocking (5 min). A total of 150 µL of a reaction cocktail containing 200 µM tris(2-carboxyethyl)phosphine (TCEP), 10 µM fluorescein *O*,*O*′-diacrylate (FOdA), and 100 mM MES (2-ethanesulfonic acid) buffer (pH 6.0) was added to 20 µL of diluted methanol supernatants in a 96-well black plate (Thermo Scientific™ Nunc™). Samples were run in duplicate. The plate was immediately covered in tinfoil and incubated at 37 °C for 30 min. The fluorescence intensity of fluorescein, formed by the interaction between selenocysteine and fluorescein *O*,*O*′-diacrylate (FOdA), was measured using a Synergy2 microplate reader (BioTek), employing an excitation wavelength of 485 nm and an emission wavelength of 535 nm. Selenocystine standards (10, 5, 2.5, 1.25, 0 µM) were prepared in 50% methanol. All standards and samples were run in duplicate. The concentration of selenocystine in our samples was quantified through extrapolation from this constructed curve and normalized to the protein content (Pierce™ BCA Protein Assay Kit, Thermo Fischer Scientific) of the respective wells to account for variations in cell quantity.

### 4.7. Immunoblotting

Protein expression was assessed via Western blot analysis. Astrocytes cultured in 6-well plates were exposed to 15 µM arsenite for 6 h, after which the arsenite-containing medium was removed and replaced with MS. After an additional 18–19 h, the cultures were washed twice with 1× HBSS (Corning Cellgro), followed by incubation with 1.6 mL of 0.05% Trypsin-EDTA (Gibco) at 37 °C for 5–10 min to aid detachment. Trypsin digestion was halted by transferring cells to an Eppendorf tube containing 1.6 mL of maintenance media-1, followed by centrifugation at 300× *g* for 5 min at 4 °C. The resulting pellet was resuspended in 3 mL of PBS and centrifuged again at 300× *g* for 5 min at 4 °C. Pellets from two wells were combined and lysed in 200 uL of RIPA lysis buffer (50 mM Tris, 1% NP-40, 1% SDS, 0.15 M NaCl, 12 mM deoxycholic acid, 5 mM iodoacetamide, 5 mM EDTA, 1× Complete Protease Inhibitor (Sigma-Aldrich) on ice for 30 min. Lysates were clarified by centrifugation (2500× *g*, 5 min, 4 °C), and the protein concentration in the supernatant was quantified using a BCA assay (Thermo Fischer Scientific). Ten micrograms of protein was denatured by boiling for 5 min in 4X SDS loading buffer (100 mM Tris, pH 6.8, 40% glycerol, 2% SDS, 50 mM EDTA, 6% β-mercaptoethanol, 0.08% bromophenol blue).

Proteins were separated by SDS-PAGE under reducing conditions and subsequently transferred onto a polyvinylidene difluoride (PVDF) membrane (0.45 um, Immobilon). Each membrane was stained with LI-COR Revert I700 to facilitate the precise quantification of protein loading in each lane for normalization purposes. After destaining, membranes were blocked in Odyssey blocking buffer (LI-COR Environmental, Lincoln, NE, USA) for 1 h at room temperature, followed by overnight incubation at 4 °C with primary antibodies against GCLc (0.93 ug/mL; rabbit polyclonal; ABclonal Technology, Woburn, MA, USA), GCL_M_ (0.38 ug/mL; rabbit polyclonal; ABclonal), and GS (0.51 ug/mL; mouse monoclonal; Novus Biologicals, Centennial, CO, USA). Species-specific secondary antibodies conjugated with spectrally distinct IRDye^®^ fluorescent dyes were used for protein detection, and images were acquired using the LI-COR ODYSSEY^®^ Fc imaging system (LI-COR). Quantitative analysis was performed using the Image Studio 3.1 software (LI-COR). The linearity of antibody–antigen interactions was confirmed to ensure that appropriate protein and antibody concentrations were used. Protein levels were normalized relative to total protein stain values.

### 4.8. Bicinchoninic Acid (BCA) Assay (Thermo Fischer Scientific)

Cell lysates/extracts were collected and loaded in 40 µL duplicates in a 96-well plate. Standards were prepared by serially diluting a 1 mg/mL sample of bovine serum albumin (BSA) in phosphate-buffered saline (PBS) and used to generate a standard curve. The assay was performed according to the manufacturer’s instructions. Absorbance was measured at 562 nm using a SpectraMax M2 plate reader. The protein content of each sample was determined by extrapolation from the standard curve based on the absorbance value of each well.

### 4.9. Statistical Analysis

All statistical analyses were performed using GraphPad Prism Version 9.4.1 or higher. Normality was assessed by the Shapiro–Wilks test. Data determined to be normal were analyzed by parametric tests: (1) one-way ANOVA followed by either Dunnett’s test for comparison to the control or Šídák’s test for all possible comparisons; or (2) two-way ANOVA followed by Fishers uncorrected LSD test for multiple comparisons. Data determined to be non-normal or those which were naturally non-normal (e.g., percentage data) were analyzed by the Kruskal–Wallis ANOVA test followed by Dunn’s test for multiple comparisons. Geometric means were used for analysis of mRNA levels. Protein levels were compared by a paired *t*-test. Statistical tests used along with the exact *p*-values are delineated in the figure legends and in the figures, respectively.

## Data Availability

Data are contained within the article and are available on request.

## Supplementary Materials

The following supporting information can be downloaded at: https://www.mdpi.com/article/10.3390/ijms26115375/s1, [83].
