# Supplementary material for: Arsenite-Mediated Transcriptional Regulation of Glutathione Synthesis in Mammalian Primary Cortical Astrocytes
Source: Int J Mol Sci. 2025 Jun 4;26(11):5375. doi: 10.3390/ijms26115375 (PMC12155450; doi:10.3390/ijms26115375)
Supplement: Supplementary file 1 [file ijms-26-05375-s001.zip › ijms-3536937-supplementary.pdf]

Supplemental Figures

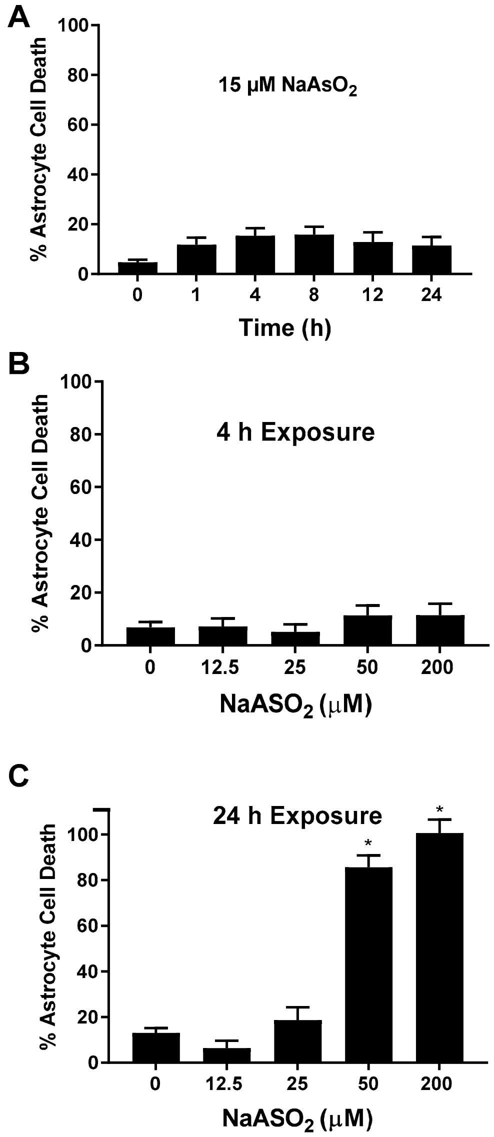

Supplemental Figure S1. ***Assessment of Arsenite Toxicity as a Function of Concentration and Exposure Time.*** Primary astrocyte cultures were treated with 15µM arsenite for increasing periods of time (n = 4 from 4 separate dissections) **(A)** or increasing concentrations of arsenite for 4 h (n = 5 from 5 separate dissections) **(B)** or 24 hr (n = 3 from two separate dissections) **(C)**, after which cell culture media was collected to measure astrocyte cell death as described [1]. Data are expressed as mean % of total astrocyte LDH (set to 100%) + SEM determined by exposing cultures to either 200µM arsenite or dichlorodiphenyltrichloroethane (DDT) for 24 h. (A, B) No significant differences were found as determined by Kruskal-Wallis ANOVA. C) Asterisk indicates values significantly different from control (0 µM) as determined by One-way ANOVA followed by Dunnet's t-test ($p < 0.0001$).

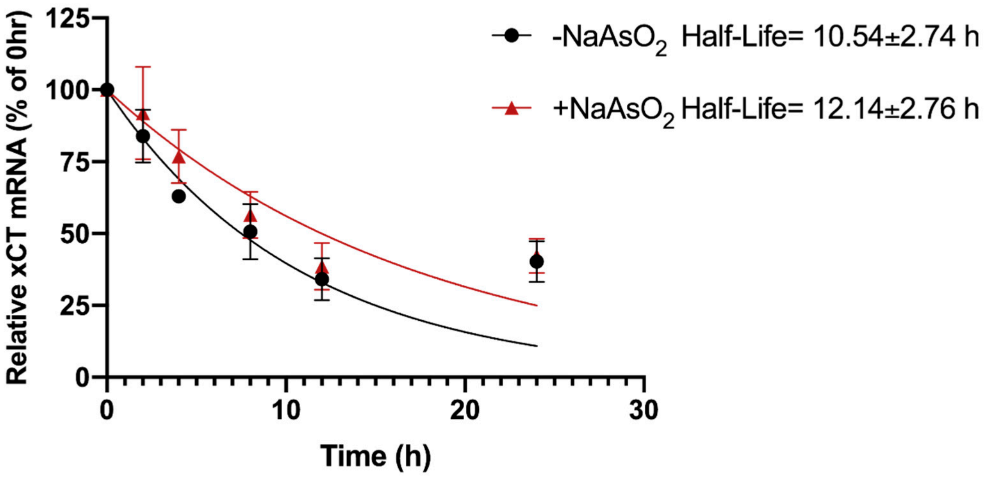

Supplemental Figure S2. ***Evaluation of Potential Post-transcriptional Regulation of xCT mRNA Expression by Arsenite.*** Primary astrocyte cultures were treated with either 15µM arsenite (NaASO2) or vehicle (control) for 8 h, after which they were washed, and 10µg/mL Act-D was added. At the times indicated, samples were harvested for RNA isolation, and relative mRNA expression of xCT was assessed via qPCR. The data are expressed as mean ± SEM scaled to zero time (both set to 100%). The mRNA half-life (t1/2) was calculated using non-linear regression analysis, specifically one-phase exponential decay. The experiment was conducted four times (n=4), with 2 to 5 cultures per time point from 5 independent astrocyte dissections. There was no significant difference in half-life between vehicle-treated and arsenite-treated cultures as determined by Mann-Whitney U test.

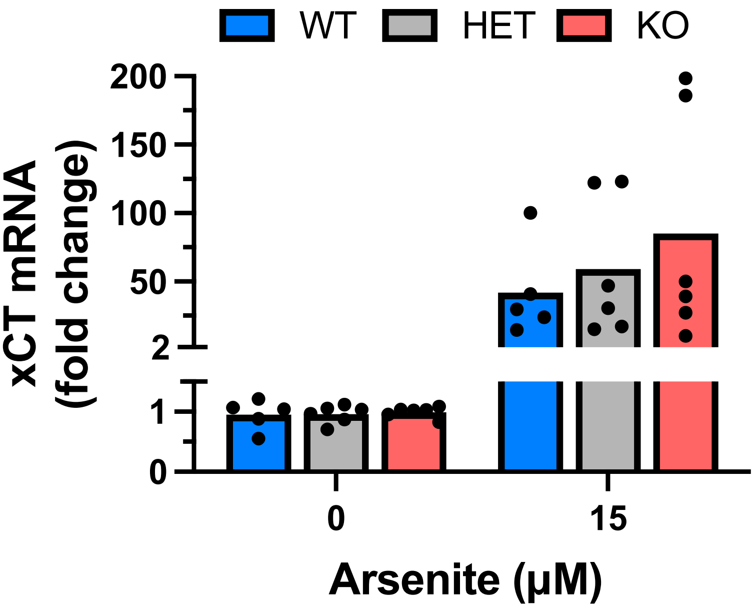

Supplemental Figure S3. ***Effect of Nrf2 on Arsenite-Induced xCT mRNA Expression.*** Nrf2 null mutant male mice (B6.129X1-Nfe2l2tm1Ywk/J; JAX stock #017009) were first bred with wildtype female mice of the same background strain (C57BL/6J; JAX stock #000664) to generate F1 Nrf2 heterozygous mice. F1 heterozygous breeding pairs were subsequently bred to obtain pups that were wildtype, heterozygous, and null for Nrf2. Primary cortical astrocytes were cultured from these mice and xCT mRNA (n=5-6 from five different dissections per genotype) was measured via RT-qPCR as described in methods. Data were normalized to each individual genotypes control (set to 1), with each dot representing an individual animal.

References

1. Uliasz, T.F.; Hewett, S.J. A Microtiter Trypan Blue Absorbance Assay for the Quantitative Determination of Excitotoxic Neuronal Injury in Cell Culture. *J Neurosci Methods* **2000**, *100*, 157–163, doi:10.1016/s0165-0270(00)00248-x.
